# PRIMEval: Optimization and screening of multiplex oligonucleotide assays

**DOI:** 10.1038/s41598-019-55883-4

**Published:** 2019-12-17

**Authors:** Rick Conzemius, Michaela Hendling, Stephan Pabinger, Ivan Barišić

**Affiliations:** Center for Health and Bioresources, AIT Austrian Institute of Technology, Giefinggasse 4, A-1210 Vienna, Austria

**Keywords:** High-throughput screening, Quality control, Software

## Abstract

The development of multiplex polymerase chain reaction and microarray assays is challenging due to primer dimer formation, unspecific hybridization events, the generation of unspecific by-products, primer depletion, and thus lower amplification efficiencies. We have developed a software workflow with three underlying algorithms that differ in their use case and specificity, allowing the complete *in silico* evaluation of such assays on user-derived data sets. We experimentally evaluated the method for the prediction of oligonucleotide hybridization events including resulting products and probes, self-dimers, cross-dimers and hairpins at different experimental conditions. The developed method allows explaining the observed artefacts through in silico WGS data and thermodynamic predictions. PRIMEval is available publicly at https://primeval.ait.ac.at.

## Introduction

The specificity of oligonucleotides is essential in nucleic acid techniques such as DNA amplification and detection technologies^1,2^. While classical microbiological methods are commonly used for the identification and characterization of pathogens, cultivation-independent genetic methods such as (real-time) polymerase chain reaction (PCR) and DNA microarrays are on the rise^3^. These methods are only cost-effective if they are highly multiplexed, which is challenging due to primer dimer formation, the formation of unwanted by-products, the resulting lower amplification efficiencies and thus, lower sensitivity due to primer depletion and accumulation of unspecific DNA^4,5^. While singleplex (e.g. Primer3, Primer-BLAST^6,7^) and multiplex (e.g. oli2go^8^) primer design tools include specificity and/or primer dimer checks, we are not aware of a software application which performs *in silico* specificity checks for combined multiplex amplification and detection assays, allows user-uploaded databases, and uses thermodynamic data to predict hybridization events including thermodynamically stable but mismatched oligonucleotides. It is crucial to consider thermodynamic data since mismatches can contribute significantly to the stabilization of DNA hybrids^9–11^. As established tools (e.g. FastPCR, MFEprimer, and Primer-BLAST^6,12,13^) either rely only on single heuristic algorithms, do not support multiplexing or do not allow combined searching for primers and probes (Supplementary Table S1), none of them covers the complete feature set. Here we present PRIMEval, a software workflow addressing these issues with multiple underlying algorithms accessible through a public web server.

## Results

**Figure 1:**
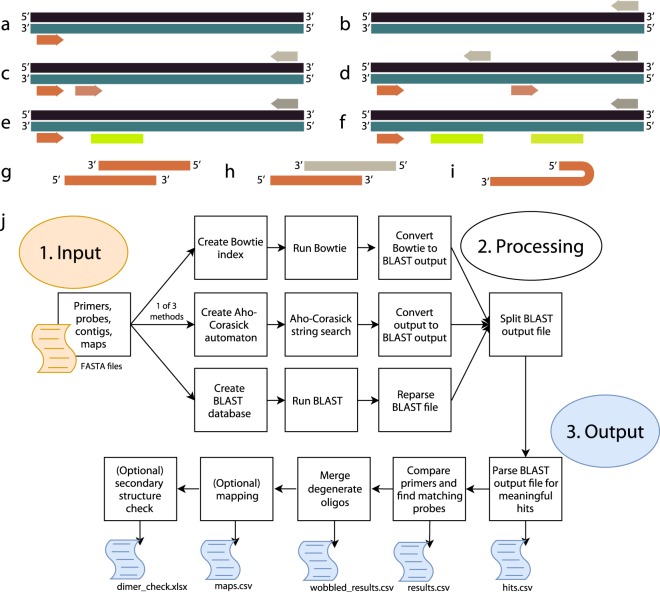
Overview of products or hits predicted by the software pipeline. (**a,b**) Forward or reverse primers binding without corresponding reverse or forward primer (no amplicon). (**c**) Multiple forward or reverse primers binding with only one corresponding reverse or forward primer (at least two amplicons). (**d**) Multiple forward and corresponding reverse primers binding in close proximity (three amplicons). (**e**) Two primers binding with one probe binding to the amplicon. (**f**) Two probes binding to the same amplicon generated by a forward and corresponding reverse primer. (**g-i**) Thermodynamic predictions of self-dimers, cross-dimers and hairpins, respectively, using the SantaLucia model with salt correction at the experimental conditions given by the user. (**j**) The general software workflow. First, an index is created by one of the three methods, then the method is executed, and the output converted to a BLAST-like output which allows using the same downstream workflow. Hits which meet the mismatch criteria are saved, then combinations of all primers in proximity (<max. product size) generate amplicons and probes binding to the amplicon are added. This is the main output, but in the next step, oligonucleotides containing degenerated bases are summarized since all three algorithms first need to resolve these bases. In the final steps, products can be mapped to user-given genes and arbitrary data, and a secondary structure check is done to predict potential self-dimers, cross-dimers and hairpins.

PRIMEval is a pipeline for the *in silico* evaluation of multiplex assays involving amplification and detection steps, hence significantly simplifying these tasks and lowering the associated costs. The software predicts all combinations of primers and probes, such as only one primer binding (Fig. 1a,b), multiple primers in one sense, but only one primer in the anti-sense (Fig. 1c), multiple sense and anti-sense primers overlapping (Fig. 1d) and single and multiple probes binding to products generated by the primers (Fig. 1e,f). Additionally, ΔG values and melting temperatures (T_m_) are reported to the user to predict self-dimers, cross-dimers and hairpins (Fig. 1g–i) in a given set of oligonucleotides. The workflow is represented in Fig. 1j. Using our server-stored databases (*i.e*. common eukaryotic model organisms), it is possible to efficiently screen a primer set for off-target hits like human background DNA.

**Figure 2:**
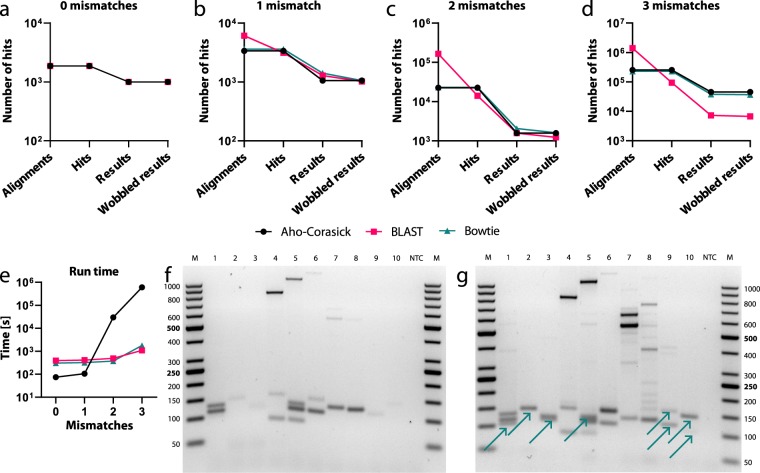
*In silico* and *in vitro* evaluation of PRIMEval. (**a–d**) The three methods (Aho-Corasick, BLAST, and Bowtie) were evaluated at their optimal parameters (Supplementary Data) and the number of hits as generated by the software is given. (**e**) The run time analysis was done at the optimal parameters for 0 to 3 mismatches. (**f,g**) The experimental evaluation of PRIMEval was done using a 45-plex PCR at 1.5 mM (**f**) and 3 mM (**g**) magnesium chloride concentration, respectively. The shown gel areas are cropped. Increasing the divalent cation concentration lowers the ΔG values, therefore the binding of the primers to their target DNA is more efficient. The emerging products, at the optimized concentration, are indicated by arrows.

We evaluated the three underlying methods for the number of retrieved alignments, hits (filtered for mismatches), corresponding results (filtered for proximity and probes) and for the special case of oligonucleotides with degenerated bases. The parameters retrieving the most hits for the different number of mismatches are used in the software workflow (Supplementary Tables S2–S6). Bowtie 1.2.2 is implemented because it is better suited for short, ungapped alignments compared to Bowtie 2^14^. If only hits without mismatches should be reported, all methods perform equally well and the non-heuristic Aho-Corasick algorithm is the fastest method. However, for 1–3 mismatches, BLAST produces a huge amount of insignificant hits, thereby increasing the computation time. The greedy Bowtie algorithm produces more significant hits than the BLAST algorithm for 1 and 2 mismatches, but less than the Aho-Corasick algorithm. With the Aho-Corasick algorithm, every reference sequence is only matched once, which is critical with degenerated oligonucleotides. At 3 mismatches, the Aho-Corasick algorithm produces the most hits, but at the huge cost of running time. Hence, we recommend using the Bowtie algorithm in most use cases or the Aho-Corasick algorithm if a small number of mismatches and no degenerate oligonucleotides are used (Fig. 2a–e).

We experimentally verified the predicted products and correlated them with the calculated nearest-neighbour thermodynamic data at low (Fig. 2f, Supplementary Table S7 and the full-length gel image in Supplementary Fig. S1) and high magnesium chloride concentration (Fig. 2g, Supplementary Table S8 and the full-length gel image in Supplementary Fig. S2). For strain 1, the product at 101 bp is missing, and in strain 2 and 3, the expected single and double bands are faint or missing. In strain 4 (at 84 and 168 bp), 5 (at 121 bp) and 6 (at 145 bp), amplicons are missing, but the by-products in these strains are correctly predicted at 823 bp, 1048 bp and 1184 bp, respectively. Strain 7 and 8 show all expected bands, while products of strain 9 and 10 are missing. Raising the magnesium chloride concentration decreases the ΔG values and increases the T_m_, hence allowing amplification of these products. From this small dataset, we observe a ΔG threshold at −10.5 kcal/mol of at least one primer (previously published at −11 kcal/mol^15^). This implies that our tool is useful both for the optimization of oligonucleotides and experimental buffer conditions.

Secondary structures are calculated using established methods and reported for ΔG values stronger than −9 kcal/mol or −5 kcal/mol for cross- or self-dimers and hairpins, respectively^7,8,13^. For the automatic screening of large datasets, users can upload a comma-separated file to match primers and internal oligonucleotides with gene names, expected product sizes and an arbitrary number of information fields (such as phenotypes or antibiotic resistance genes). PRIMEval can also be used to extract sequence data and to determine the position of target sequences in genomes.

## Discussion

PRIMEval was tested and used by several members of our research unit over the last two years for the optimization of multiplex assays. It helped significantly to reduce evaluation costs, to explain unclear observations and to facilitate decisions on how to optimize multiplex oligonucleotide sets. Many *in vitro* observed artefacts can be explained through *in silico* WGS data and thermodynamic predictions. The incorporated methods were evaluated, and the highest recovery rates were obtained using the Bowtie algorithm or the Aho-Corasick algorithm if the oligonucleotides do not contain degenerated bases. Other methods such as BIGSI are currently not suited because reconstructions from such data structures are not possible and coverage information is not stored^16^. Therefore, the implemented string searching algorithms are the limiting factors and it would not be possible to screen efficiently using thermodynamic data only (*i.e*. with unlimited mismatches) on huge datasets. Also at the current stage, the mismatch positions in oligonucleotides are visualized and left open to user interpretation as published methods are not clearly interpretable^11,17,18^. Therefore, we included thermodynamic data as *e.g*. G-G mismatches can contribute up to −2.2 kcal/mol to the stabilization of the duplex whereas other mismatches can be highly destabilizing^9,10^. The possibility of using user-defined reference sequences is currently unique and user-defined mapping files allow the application of PRIMEval for many use cases (*e.g*. screening for antibiotic resistance genes or virulence factors).

## Methods

### Input

The web service allows the user to upload own reference sequences, primer sets and probe (internal oligonucleotide) sets in the FASTA format. Matching probes can be marked by a correct filename terminology. Alternatively, users can select server-stored Bowtie indices of common eukaryotic model organisms instead of uploading own sequences. The user selects the underlying search algorithm, a maximum number of mismatches in primers and probes, and a maximum product length. For thermodynamic analyses and for optional secondary structure checks, annealing temperatures and salt concentrations must be indicated for the amplification and hybridization steps (Supplementary Fig. S3).

### Searching algorithms

Oligonucleotides are mapped to the reference sequences using BLAST+ 2.7.1^19,20^, Bowtie 1.2.2^21^ or the Aho-Corasick algorithm^22^. BLAST (qcov_hsp_perc, perc_identity, word_size), Bowtie (seedlen, maqerr, seedmms) and Aho-Corasick were evaluated experimentally for different parameters in terms of run time and the number of retrieved hits on four 45-plex primer sets (Supplementary Tables S9-S12) targeting antibiotic resistances on 91 bacterial genomes (Supplementary Table S13).

### Predicting PCR products

The hits obtained from the different algorithms are converted to the same file format in order to use the same downstream pipeline. Hits meeting the mismatch criteria are checked against each other for proximity (product length). Primer pairs are created if the hits are in accordance with the described criteria. If one or more probes fall into the amplicon region, the probes are added to the oligonucleotide pair.

### Secondary structure check

Hairpins and cross-dimers of all sequences from each oligonucleotide set are predicted using a Python implementation of the primer3 core (primer3-py). SantaLucia’s model using salt correction is used to predict T_m_ and ΔG values^10^.

### Output

A visual representation of primers, probes, and products allows an intuitive inspection whether primers might overlap or multiple probes binding to an amplicon. Lists of matching primers including probes are given as well as single hits alone (e.g. single primer, probe without primers, etc.). Dimer checks are represented as heat maps and possible candidates reported if ΔG ≤ −9 kcal/mol or T_m_ + 3 ≥ step T_m_ for hairpins. All files can be downloaded in the CSV file format including a summary of oligonucleotides containing degenerated bases. These files include the contig positions, oligonucleotide set, product sizes, the number of mismatches and representations thereof, ΔG and Tm values for oligonucleotides, the T_m_ of the product and the product itself (Supplementary Fig. S4).

### Implementation

The server runs on Ubuntu 16.04 LTS on a machine containing four 16-core processors and 384 GB RAM. The applications are written in Python 3.4.3 and additionally make use of Biopython^23^, Pandas^24^, and the Redis/RQ queue management system.

### Experimental evaluation

A 45-plex set of primers (Supplementary Table S14) targeting antibiotic resistance genes was experimentally evaluated on ten sequenced clinical bacterial strains and correlated with the *in silico* data. The reaction mixture (20 µl) comprised Molzym PCR buffer (1.5 mM MgCl_2_), 200 µM of each dNTP, 1 unit of Hot MolTaq DNA-free polymerase (Molzym), 111 nM of each primer, 10 ng of target DNA and additionally 1.5 mM MgCl_2_ in a second PCR for comparison. The reaction was incubated as follows: 5 min at 94 °C, 30 cycles of 30 s each at 94 °C, 55 °C and 72 °C, and 7 min at 72 °C. The products were separated and visualized on a 2% agarose gel. Brightness and contrast were adjusted, and the gel image was inverted using ImageJ.

## Supplementary information

Supplementary Information

## Data Availability

The code implementing the main method can be found at https://github.com/rczms/primeval and is under the MIT license. The public PRIMEval web server is available under https://primeval.ait.ac.at/.
