# Supplementary material for: PRIMEval: Optimization and screening of multiplex oligonucleotide assays
Source: Sci Rep. 2019 Dec 17;9:19286. doi: 10.1038/s41598-019-55883-4 (PMC6917790; doi:10.1038/s41598-019-55883-4)
Supplement: Supplementary file 1 — Supplementary Information [file 41598_2019_55883_MOESM1_ESM.pdf]

PRIMEval: Optimization and screening of multiplex oligonucleotide assays

Supplementary Information

Rick Conzemius, Michaela Hendling, Stephan Pabinger and Ivan Barišić

Supplementary Table S1: List of available software tools for the evaluation of oligonucleotides. Y marks supported features, (Y) marks partially supported features and N marks unsupported features.

Method	Reference	Primers	Internal oligo	Oligonucleotide combinations	Multiple algorithms	Multiplexing	Mismatches	Mapping	Degenerated oligonucleotides	User-defined DB	Pre-defined DB	Dimers	Hairpins	Thermodynamic data	Product
**PRIMEval**		**Y**	**Y**	**Y**	**Y**	**Y**	**Y**	**Y**	**Y**	**Y**	**Y**	**Y**	**Y**	**Y**	**Y**
PrimerDimer	(1)	Y	N	Y	N	Y	N	N	N	N	N	Y	N	N	N
PrimerROC	(2)	Y	N	N	N	Y	N	N	N	N	N	Y	N	N	N
FastPCR	(3)	Y	(Y)	(Y)	N	(Y)	Y	N	Y	(Y)	N	N	N	N	(Y)
MFEprimer	(4)	Y	N	Y	N	(Y)	(Y)	N	Y	N	Y	Y	Y	Y	Y
Primer-BLAST	(5)	Y	N	N	N	N	Y	N	N	Y	Y	Y	Y	Y	Y
UCSC In-silico PCR	(6)	Y	N	N	N	N	Y	N	N	N	Y	N	N	N	Y
jPCR	(7)	Y	N	(Y)	N	(Y)	Y	N	Y	Y	N	Y	Y	N	Y
PROBE	(8)	N	Y	N	N	N	Y	N	N	Y	N	N	N	N	N
AutoDimer	(9)	Y	Y	Y	N	Y	N	N	N	N	N	Y	Y	Y	N
Simulate_PCR	(10)	Y	Y	Y	N	Y	Y	N	Y	Y	N	N	N	N	Y

Supplementary References

1. Lu J, Johnston A, Berichon P, Ru K-l, Korbie D, Trau M. PrimerSuite: A High-Throughput Web-Based Primer Design Program for Multiplex Bisulfite PCR. Sci Rep 2017. doi:10.1038/srep41328.
2. Johnston A d., Lu J, Ru K-l, Korbie D, Trau M. PrimerROC: accurate condition-independent dimer prediction using ROC analysis. Sci Rep 2019. doi:10.1038/s41598-018-36612-9.
3. Kalendar R, Khassenov B, Ramankulov Y, Samuilova O, Ivanov KI. FastPCR: An in silico tool for fast primer and probe design and advanced sequence analysis. Genomics. 2017;109:312–9. doi:10.1016/j.ygeno.2017.05.005.
4. Qu W, Zhang C. Selecting specific PCR primers with MFEprimer. Methods Mol Biol. 2015;1275:201–13. doi:10.1007/978-1-4939-2365-6_15.
5. Ye J, Coulouris G, Zaretskaya I, Cutcutache I, Rozen S, Madden TL. Primer-BLAST: a tool to design target-specific primers for polymerase chain reaction. BMC Bioinformatics. 2012;13:134. doi:10.1186/1471-2105-13-134.
6. Yu B, Zhang C. In silico PCR analysis. Methods Mol Biol. 2011;760:91–107. doi:10.1007/978-1-61779-176-5_6.
7. Kalendar R, Lee D, Schulman AH. Java web tools for PCR, in silico PCR, and oligonucleotide assembly and analysis. Genomics. 2011;98:137–44. doi:10.1016/j.ygeno.2011.04.009.
8. Pozhitkov AE, Tautz D. An algorithm and program for finding sequence specific oligonucleotide probes for species identification. BMC Bioinformatics. 2002;3:9.
9. Vallone PM, Butler JM. AutoDimer: a screening tool for primer-dimer and hairpin structures. BioTechniques. 2004;37:226–31. doi:10.2144/04372ST03.
10. Gardner SN, Slezak T. Simulate_PCR for amplicon prediction and annotation from multiplex, degenerate primers and probes. BMC Bioinformatics. 2014;15:237. doi:10.1186/1471-2105-15-237.

Supplementary Table S2: The parameters used in the searching algorithms.

Method	Parameters
Aho-Corasick	No parameterization.
BLAST+ 2.7.1	`makeblastdb -in contigs.fasta -dbtype nucl -out blastdb`<br><br>`blastn -db blastdb -query input.fasta -out blast_results.txt -outfmt "6 qseqid sseqid nident qlen length mismatch qstart qend sstart sseq sstrand send" -num_threads " $threads -evalue 200000 -qcov_hsp_perc $qcov -perc_identity $perciden -max_target_seqs 2000000 -word_size 4 –ungapped`<br><br>`For 0 mismatches: $qcov = 100, $perciden = 100`<br>`For 1 mismatch:   $qcov =  90, $perciden =  90`<br>`For 2 mismatches: $qcov =  85, $perciden =  85`<br>`For 3 mismatches: $qcov =  80, $perciden =  80`
Bowtie 1.2.2	`bowtie-build --threads $threads -f contigs.fasta bowtie_index`<br><br>`bowtie -f -a -p $threads -n $mm -l $l -e $e bowtie_index input.fasta bowtie_results.fasta`<br><br>`For 0 mismatches: $mm = 0, $l = 5, $e = 10`<br>`For 1 mismatch:   $mm = 1, $l = 5, $e = 40`<br>`For 2 mismatches: $mm = 2, $l = 5, $e = 70`<br>`For 3 mismatches: $mm = 3, $l = 5, $e = 100`

Supplementary Table S3: Parameter evaluation if no mismatches are allowed.

Method	Parameter 1	Parameter 2	Alignments	Hits	Results	Wobbled results	Run time [s]
Aho-Corasick			1870	1870	998	998	71
Bowtie	l = 5	e = 10	1870	1870	998	998	313
Bowtie	l = 5	e = 40	3048	1870	998	998	313
Bowtie	l = 5	e = 70	12635	1870	998	998	312
Bowtie	l = 5	e = 100	91793	1870	998	998	314
Bowtie	l = 5	e = 130	552176	1870	998	998	318
Bowtie	l = 5	e = 160	2598448	1870	998	998	339
Bowtie	l = 9	e = 10	1870	1870	998	998	313
Bowtie	l = 9	e = 40	2661	1870	998	998	313
Bowtie	l = 9	e = 70	6925	1870	998	998	312
Bowtie	l = 9	e = 100	28663	1870	998	998	313
Bowtie	l = 9	e = 130	102724	1870	998	998	314
Bowtie	l = 9	e = 160	276193	1870	998	998	316
Bowtie	l = 13	e = 10	1870	1870	998	998	312
Bowtie	l = 13	e = 40	2296	1870	998	998	313
Bowtie	l = 13	e = 70	3225	1870	998	998	314
Bowtie	l = 13	e = 100	5138	1870	998	998	313
Bowtie	l = 13	e = 130	7191	1870	998	998	313
Bowtie	l = 13	e = 160	8440	1870	998	998	312
Bowtie	l = 17	e = 10	1870	1870	998	998	312
Bowtie	l = 17	e = 40	1917	1870	998	998	312
Bowtie	l = 17	e = 70	1927	1870	998	998	313
Bowtie	l = 17	e = 100	1931	1870	998	998	311
Bowtie	l = 17	e = 130	1933	1870	998	998	312
Bowtie	l = 17	e = 160	1935	1870	998	998	312
Bowtie	l = 21	e = 10	1870	1870	998	998	312
Bowtie	l = 21	e = 40	1882	1870	998	998	313
Bowtie	l = 21	e = 70	1883	1870	998	998	311
Bowtie	l = 21	e = 100	1883	1870	998	998	312
Bowtie	l = 21	e = 130	1883	1870	998	998	313
Bowtie	l = 21	e = 160	1883	1870	998	998	312
Bowtie	l = 25	e = 10	1870	1870	998	998	312
Bowtie	l = 25	e = 40	1870	1870	998	998	311
Bowtie	l = 25	e = 70	1870	1870	998	998	312
Bowtie	l = 25	e = 100	1870	1870	998	998	313
Bowtie	l = 25	e = 130	1870	1870	998	998	312
Bowtie	l = 25	e = 160	1870	1870	998	998	313
Bowtie	l = 28	e = 10	1870	1870	998	998	312
Bowtie	l = 28	e = 40	1870	1870	998	998	311
Bowtie	l = 28	e = 70	1870	1870	998	998	311
Bowtie	l = 28	e = 100	1870	1870	998	998	312
Bowtie	l = 28	e = 130	1870	1870	998	998	312
Bowtie	l = 28	e = 160	1870	1870	998	998	311
BLAST	qcov = 75	perc = 75	3033244	1870	998	998	1150

BLAST	qcov = 75	perc = 80	2963619	1870	998	998	1127
BLAST	qcov = 75	perc = 85	1047317	1870	998	998	735
BLAST	qcov = 75	perc = 90	257800	1870	998	998	515
BLAST	qcov = 75	perc = 95	15782	1870	998	998	413
BLAST	qcov = 75	perc = 100	15683	1870	998	998	384
BLAST	qcov = 80	perc = 75	1466503	1870	998	998	839
BLAST	qcov = 80	perc = 80	1404308	1870	998	998	797
BLAST	qcov = 80	perc = 85	557315	1870	998	998	554
BLAST	qcov = 80	perc = 90	67226	1870	998	998	416
BLAST	qcov = 80	perc = 95	5174	1870	998	998	395
BLAST	qcov = 80	perc = 100	5075	1870	998	998	383
BLAST	qcov = 85	perc = 75	736644	1870	998	998	630
BLAST	qcov = 85	perc = 80	682335	1870	998	998	579
BLAST	qcov = 85	perc = 85	164755	1870	998	998	444
BLAST	qcov = 85	perc = 90	18590	1870	998	998	405
BLAST	qcov = 85	perc = 95	2805	1870	998	998	413
BLAST	qcov = 85	perc = 100	2716	1870	998	998	370
BLAST	qcov = 90	perc = 75	262655	1870	998	998	509
BLAST	qcov = 90	perc = 80	218176	1870	998	998	457
BLAST	qcov = 90	perc = 85	43821	1870	998	998	430
BLAST	qcov = 90	perc = 90	6120	1870	998	998	368
BLAST	qcov = 90	perc = 95	2150	1870	998	998	367
BLAST	qcov = 90	perc = 100	2077	1870	998	998	413
BLAST	qcov = 95	perc = 75	104038	1870	998	998	406
BLAST	qcov = 95	perc = 80	66171	1870	998	998	409
BLAST	qcov = 95	perc = 85	13444	1870	998	998	395
BLAST	qcov = 95	perc = 90	3228	1870	998	998	386
BLAST	qcov = 95	perc = 95	1944	1870	998	998	409
BLAST	qcov = 95	perc = 100	1886	1870	998	998	383
BLAST	qcov = 100	perc = 75	71760	1870	998	998	424
BLAST	qcov = 100	perc = 80	52416	1870	998	998	430
BLAST	qcov = 100	perc = 85	11174	1870	998	998	403
BLAST	qcov = 100	perc = 90	3016	1870	998	998	384
BLAST	qcov = 100	perc = 95	1920	1870	998	998	387
BLAST	qcov = 100	perc = 100	1870	1870	998	998	398

Supplementary Table S4: Parameter evaluation if 1 mismatch is allowed.

Method	Parameter 1	Parameter 2	Alignments	Hits	Results	Wobbled results	Run time [s]
Aho-Corasick			3371	3371	1056	1056	111
Bowtie	l = 5	e = 10	1870	1870	998	998	315
Bowtie	l = 5	e = 40	3622	3622	1412	1056	320
Bowtie	l = 5	e = 70	21504	3622	1412	1056	319
Bowtie	l = 5	e = 100	210481	3622	1412	1056	320
Bowtie	l = 5	e = 130	1619259	3622	1412	1056	338
Bowtie	l = 5	e = 160	9484654	3622	1412	1056	428
Bowtie	l = 9	e = 10	1870	1870	998	998	313
Bowtie	l = 9	e = 40	3622	3622	1412	1056	318
Bowtie	l = 9	e = 70	17904	3622	1412	1056	320
Bowtie	l = 9	e = 100	125970	3622	1412	1056	321
Bowtie	l = 9	e = 130	664113	3622	1412	1056	327
Bowtie	l = 9	e = 160	2511884	3622	1412	1056	345
Bowtie	l = 13	e = 10	1870	1870	998	998	314
Bowtie	l = 13	e = 40	3622	3622	1412	1056	320
Bowtie	l = 13	e = 70	11744	3622	1412	1056	320
Bowtie	l = 13	e = 100	42151	3622	1412	1056	323
Bowtie	l = 13	e = 130	104319	3622	1412	1056	320
Bowtie	l = 13	e = 160	171287	3622	1412	1056	322
Bowtie	l = 17	e = 10	1870	1870	998	998	315
Bowtie	l = 17	e = 40	3622	3622	1412	1056	319
Bowtie	l = 17	e = 70	4063	3622	1412	1056	322
Bowtie	l = 17	e = 100	4346	3622	1412	1056	319
Bowtie	l = 17	e = 130	4618	3622	1412	1056	320
Bowtie	l = 17	e = 160	4877	3622	1412	1056	319
Bowtie	l = 21	e = 10	1870	1870	998	998	315
Bowtie	l = 21	e = 40	3622	3622	1412	1056	319
Bowtie	l = 21	e = 70	3637	3622	1412	1056	322
Bowtie	l = 21	e = 100	3639	3622	1412	1056	318
Bowtie	l = 21	e = 130	3639	3622	1412	1056	319
Bowtie	l = 21	e = 160	3640	3622	1412	1056	318
Bowtie	l = 25	e = 10	1870	1870	998	998	314
Bowtie	l = 25	e = 40	3622	3622	1412	1056	318
Bowtie	l = 25	e = 70	3622	3622	1412	1056	319
Bowtie	l = 25	e = 100	3622	3622	1412	1056	319
Bowtie	l = 25	e = 130	3622	3622	1412	1056	319
Bowtie	l = 25	e = 160	3622	3622	1412	1056	320
Bowtie	l = 28	e = 10	1870	1870	998	998	313
Bowtie	l = 28	e = 40	3622	3622	1412	1056	318
Bowtie	l = 28	e = 70	3622	3622	1412	1056	320
Bowtie	l = 28	e = 100	3622	3622	1412	1056	319
Bowtie	l = 28	e = 130	3622	3622	1412	1056	322
Bowtie	l = 28	e = 160	3622	3622	1412	1056	318
BLAST	qcov = 75	perc = 75	3033244	3123	1284	1021	1113

BLAST	qcov = 75	perc = 80	2963619	3123	1284	1021	1110
BLAST	qcov = 75	perc = 85	1047317	3123	1284	1021	680
BLAST	qcov = 75	perc = 90	257800	3123	1284	1021	504
BLAST	qcov = 75	perc = 95	15782	2109	1029	1009	402
BLAST	qcov = 75	perc = 100	15683	2059	1024	1004	405
BLAST	qcov = 80	perc = 75	1466503	3123	1284	1021	817
BLAST	qcov = 80	perc = 80	1404308	3123	1284	1021	805
BLAST	qcov = 80	perc = 85	557315	3123	1284	1021	607
BLAST	qcov = 80	perc = 90	67226	3123	1284	1021	417
BLAST	qcov = 80	perc = 95	5174	2109	1029	1009	397
BLAST	qcov = 80	perc = 100	5075	2059	1024	1004	406
BLAST	qcov = 85	perc = 75	736644	3123	1284	1021	644
BLAST	qcov = 85	perc = 80	682335	3123	1284	1021	571
BLAST	qcov = 85	perc = 85	164755	3123	1284	1021	470
BLAST	qcov = 85	perc = 90	18590	3123	1284	1021	399
BLAST	qcov = 85	perc = 95	2805	2109	1029	1009	415
BLAST	qcov = 85	perc = 100	2716	2059	1024	1004	391
BLAST	qcov = 90	perc = 75	262655	3123	1284	1021	513
BLAST	qcov = 90	perc = 80	218176	3123	1284	1021	462
BLAST	qcov = 90	perc = 85	43821	3123	1284	1021	442
BLAST	qcov = 90	perc = 90	6120	3123	1284	1021	404
BLAST	qcov = 90	perc = 95	2150	2109	1029	1009	422
BLAST	qcov = 90	perc = 100	2077	2059	1024	1004	376
BLAST	qcov = 95	perc = 75	104038	2950	1261	1018	445
BLAST	qcov = 95	perc = 80	66171	2950	1261	1018	442
BLAST	qcov = 95	perc = 85	13444	2950	1261	1018	413
BLAST	qcov = 95	perc = 90	3228	2950	1261	1018	403
BLAST	qcov = 95	perc = 95	1944	1936	1007	1007	407
BLAST	qcov = 95	perc = 100	1886	1886	1002	1002	379
BLAST	qcov = 100	perc = 75	71760	2934	1257	1014	400
BLAST	qcov = 100	perc = 80	52416	2934	1257	1014	414
BLAST	qcov = 100	perc = 85	11174	2934	1257	1014	417
BLAST	qcov = 100	perc = 90	3016	2934	1257	1014	406
BLAST	qcov = 100	perc = 95	1920	1920	1003	1003	414
BLAST	qcov = 100	perc = 100	1870	1870	998	998	373

Supplementary Table S5: Parameter evaluation if 2 mismatches are allowed.

Method	Parameter 1	Parameter 2	Alignments	Hits	Results	Wobbled results	Run time [s]
Aho-Corasick			22510	22510	1569	1569	30799
Bowtie	l = 5	e = 10	1870	1870	998	998	312
Bowtie	l = 5	e = 40	3622	3622	1412	1056	318
Bowtie	l = 5	e = 70	23101	23101	2058	1614	365
Bowtie	l = 5	e = 100	229575	22186	2025	1590	361
Bowtie	l = 5	e = 130	1868558	22123	2022	1587	385
Bowtie	l = 5	e = 160	11780127	22123	2022	1587	554
Bowtie	l = 9	e = 10	1870	1870	998	998	313
Bowtie	l = 9	e = 40	3622	3622	1412	1056	323
Bowtie	l = 9	e = 70	22169	22169	1982	1568	357
Bowtie	l = 9	e = 100	175665	20474	1886	1484	358
Bowtie	l = 9	e = 130	1104436	20372	1881	1479	373
Bowtie	l = 9	e = 160	5105932	20361	1881	1479	430
Bowtie	l = 13	e = 10	1870	1870	998	998	315
Bowtie	l = 13	e = 40	3622	3622	1412	1056	319
Bowtie	l = 13	e = 70	19025	19025	1793	1395	352
Bowtie	l = 13	e = 100	103100	17705	1750	1356	350
Bowtie	l = 13	e = 130	376594	17463	1726	1338	353
Bowtie	l = 13	e = 160	895368	17403	1723	1335	361
Bowtie	l = 17	e = 10	1870	1870	998	998	315
Bowtie	l = 17	e = 40	3622	3622	1412	1056	319
Bowtie	l = 17	e = 70	16175	16175	1767	1344	344
Bowtie	l = 17	e = 100	22104	16160	1767	1344	345
Bowtie	l = 17	e = 130	25685	16158	1767	1344	345
Bowtie	l = 17	e = 160	28266	16157	1766	1343	346
Bowtie	l = 21	e = 10	1870	1870	998	998	314
Bowtie	l = 21	e = 40	3622	3622	1412	1056	319
Bowtie	l = 21	e = 70	16041	16041	1738	1313	344
Bowtie	l = 21	e = 100	16079	16041	1738	1313	342
Bowtie	l = 21	e = 130	16097	16041	1738	1313	344
Bowtie	l = 21	e = 160	16104	16041	1738	1313	346
Bowtie	l = 25	e = 10	1870	1870	998	998	315
Bowtie	l = 25	e = 40	3622	3622	1412	1056	319
Bowtie	l = 25	e = 70	16039	16039	1739	1314	343
Bowtie	l = 25	e = 100	16039	16039	1739	1314	343
Bowtie	l = 25	e = 130	16039	16039	1739	1314	347
Bowtie	l = 25	e = 160	16039	16039	1739	1314	344
Bowtie	l = 28	e = 10	1870	1870	998	998	314
Bowtie	l = 28	e = 40	3622	3622	1412	1056	319
Bowtie	l = 28	e = 70	16039	16039	1739	1314	345
Bowtie	l = 28	e = 100	16039	16039	1739	1314	345
Bowtie	l = 28	e = 130	16039	16039	1739	1314	344
Bowtie	l = 28	e = 160	16039	16039	1739	1314	349
BLAST	qcov = 75	perc = 75	3033244	14044	1570	1230	1214

BLAST	qcov = 75	perc = 80	2963619	14044	1570	1230	1151
BLAST	qcov = 75	perc = 85	1047317	14044	1570	1230	752
BLAST	qcov = 75	perc = 90	257800	6405	1372	1071	524
BLAST	qcov = 75	perc = 95	15782	2741	1055	1018	406
BLAST	qcov = 75	perc = 100	15683	2683	1049	1012	404
BLAST	qcov = 80	perc = 75	1466503	14044	1570	1230	838
BLAST	qcov = 80	perc = 80	1404308	14044	1570	1230	868
BLAST	qcov = 80	perc = 85	557315	14044	1570	1230	591
BLAST	qcov = 80	perc = 90	67226	6405	1372	1071	445
BLAST	qcov = 80	perc = 95	5174	2741	1055	1018	376
BLAST	qcov = 80	perc = 100	5075	2683	1049	1012	383
BLAST	qcov = 85	perc = 75	736644	14044	1570	1230	676
BLAST	qcov = 85	perc = 80	682335	14044	1570	1230	644
BLAST	qcov = 85	perc = 85	164755	14044	1570	1230	485
BLAST	qcov = 85	perc = 90	18590	6405	1372	1071	421
BLAST	qcov = 85	perc = 95	2805	2741	1055	1018	382
BLAST	qcov = 85	perc = 100	2716	2683	1049	1012	406
BLAST	qcov = 90	perc = 75	262655	13438	1514	1212	526
BLAST	qcov = 90	perc = 80	218176	13438	1514	1212	493
BLAST	qcov = 90	perc = 85	43821	13438	1514	1212	446
BLAST	qcov = 90	perc = 90	6120	5799	1325	1061	418
BLAST	qcov = 90	perc = 95	2150	2135	1032	1013	382
BLAST	qcov = 90	perc = 100	2077	2077	1026	1007	385
BLAST	qcov = 95	perc = 75	104038	10784	1378	1131	469
BLAST	qcov = 95	perc = 80	66171	10784	1378	1131	449
BLAST	qcov = 95	perc = 85	13444	10784	1378	1131	413
BLAST	qcov = 95	perc = 90	3228	3145	1266	1024	368
BLAST	qcov = 95	perc = 95	1944	1944	1007	1008	398
BLAST	qcov = 95	perc = 100	1886	1886	1002	1003	395
BLAST	qcov = 100	perc = 75	71760	10655	1373	1126	452
BLAST	qcov = 100	perc = 80	52416	10655	1373	1126	453
BLAST	qcov = 100	perc = 85	11174	10655	1373	1126	427
BLAST	qcov = 100	perc = 90	3016	3016	1261	1019	407
BLAST	qcov = 100	perc = 95	1920	1920	1003	1004	479
BLAST	qcov = 100	perc = 100	1870	1870	998	999	382

Supplementary Table S6: Parameter evaluation if 3 mismatches are allowed.

Method	Parameter 1	Parameter 2	Alignments	Hits	Results	Wobbled results	Run time [s]
Aho-Corasick			252578	252578	45240	45240	593475
Bowtie	l = 5	e = 10	1870	1870	998	998	339
Bowtie	l = 5	e = 40	3622	3622	1412	1056	345
Bowtie	l = 5	e = 70	23101	23101	2058	1614	390
Bowtie	l = 5	e = 100	229779	229779	38072	36765	1760
Bowtie	l = 5	e = 130	1878773	227903	37517	36217	1630
Bowtie	l = 5	e = 160	11934707	227838	37501	36201	2658
Bowtie	l = 9	e = 10	1870	1870	998	998	340
Bowtie	l = 9	e = 40	3622	3622	1412	1056	345
Bowtie	l = 9	e = 70	21907	21907	1972	1560	382
Bowtie	l = 9	e = 100	180867	180867	23563	22632	1275
Bowtie	l = 9	e = 130	1210394	178579	23001	22082	1226
Bowtie	l = 9	e = 160	6124276	178351	22954	22035	1444
Bowtie	l = 13	e = 10	1870	1870	998	998	343
Bowtie	l = 13	e = 40	3622	3622	1412	1056	348
Bowtie	l = 13	e = 70	18906	18906	1792	1394	378
Bowtie	l = 13	e = 100	121928	121928	11796	11161	731
Bowtie	l = 13	e = 130	595538	120011	11459	10827	794
Bowtie	l = 13	e = 160	1961450	119650	11376	10746	844
Bowtie	l = 17	e = 10	1870	1870	998	998	341
Bowtie	l = 17	e = 40	3622	3622	1412	1056	345
Bowtie	l = 17	e = 70	15898	15898	1748	1325	371
Bowtie	l = 17	e = 100	71509	71509	4970	4486	527
Bowtie	l = 17	e = 130	100436	71532	4966	4483	519
Bowtie	l = 17	e = 160	119709	71506	4966	4483	523
Bowtie	l = 21	e = 10	1870	1870	998	998	340
Bowtie	l = 21	e = 40	3622	3622	1412	1056	345
Bowtie	l = 21	e = 70	15764	15764	1716	1293	369
Bowtie	l = 21	e = 100	68819	68819	4717	4250	510
Bowtie	l = 21	e = 130	69009	68819	4717	4250	512
Bowtie	l = 21	e = 160	69101	68819	4717	4250	518
Bowtie	l = 25	e = 10	1870	1870	998	998	339
Bowtie	l = 25	e = 40	3622	3622	1412	1056	345
Bowtie	l = 25	e = 70	15758	15758	1717	1294	371
Bowtie	l = 25	e = 100	68790	68790	4717	4250	520
Bowtie	l = 25	e = 130	68790	68790	4717	4250	529
Bowtie	l = 25	e = 160	68790	68790	4717	4250	523
Bowtie	l = 28	e = 10	1870	1870	998	998	339
Bowtie	l = 28	e = 40	3622	3622	1412	1056	343
Bowtie	l = 28	e = 70	15758	15758	1717	1294	369
Bowtie	l = 28	e = 100	68790	68790	4717	4250	518
Bowtie	l = 28	e = 130	68790	68790	4717	4250	515
Bowtie	l = 28	e = 160	68790	68790	4717	4250	536
BLAST	qcov = 75	perc = 75	3033244	94008	7273	6718	1576

BLAST	qcov = 75	perc = 80	2963619	94008	7273	6718	1613
BLAST	qcov = 75	perc = 85	1047317	55290	3461	3009	954
BLAST	qcov = 75	perc = 90	257800	20280	1740	1373	623
BLAST	qcov = 75	perc = 95	15782	5101	1145	1061	448
BLAST	qcov = 75	perc = 100	15683	5028	1132	1048	470
BLAST	qcov = 80	perc = 75	1466503	94008	7273	6718	1179
BLAST	qcov = 80	perc = 80	1404308	94008	7273	6718	1093
BLAST	qcov = 80	perc = 85	557315	55290	3461	3009	729
BLAST	qcov = 80	perc = 90	67226	20280	1740	1373	463
BLAST	qcov = 80	perc = 95	5174	5101	1145	1061	427
BLAST	qcov = 80	perc = 100	5075	5028	1132	1048	414
BLAST	qcov = 85	perc = 75	736644	91696	6952	6426	878
BLAST	qcov = 85	perc = 80	682335	91696	6952	6426	871
BLAST	qcov = 85	perc = 85	164755	52978	3261	2833	598
BLAST	qcov = 85	perc = 90	18590	17968	1640	1292	440
BLAST	qcov = 85	perc = 95	2805	2789	1085	1019	397
BLAST	qcov = 85	perc = 100	2716	2716	1079	1013	403
BLAST	qcov = 90	perc = 75	262655	79689	5511	5114	671
BLAST	qcov = 90	perc = 80	218176	79689	5511	5114	661
BLAST	qcov = 90	perc = 85	43821	40971	2410	2089	479
BLAST	qcov = 90	perc = 90	6120	5961	1344	1080	420
BLAST	qcov = 90	perc = 95	2150	2150	1032	1013	388
BLAST	qcov = 90	perc = 100	2077	2077	1026	1007	394
BLAST	qcov = 95	perc = 75	104038	51368	3004	2714	545
BLAST	qcov = 95	perc = 80	66171	51368	3004	2714	522
BLAST	qcov = 95	perc = 85	13444	12650	1392	1145	432
BLAST	qcov = 95	perc = 90	3228	3228	1266	1024	413
BLAST	qcov = 95	perc = 95	1944	1944	1007	1008	399
BLAST	qcov = 95	perc = 100	1886	1886	1002	1003	421
BLAST	qcov = 100	perc = 75	71760	49892	2952	2671	487
BLAST	qcov = 100	perc = 80	52416	49892	2952	2671	505
BLAST	qcov = 100	perc = 85	11174	11174	1375	1128	439
BLAST	qcov = 100	perc = 90	3016	3016	1261	1019	382
BLAST	qcov = 100	perc = 95	1920	1920	1003	1004	397
BLAST	qcov = 100	perc = 100	1870	1870	998	999	398

Supplementary Table S7: Predicted products and thermodynamic data at 1.5 mM $MgCl_2$. Original data of the PRIMEval run at max. 1 mismatch can be found under https://primeval.ait.ac.at/run/1043325579.

Primer 1	Primer 2	Size	MM 1	MM 2	Primer 1 Tm (°C)	Primer 2 Tm (°C)	Primer 1 ΔG (kcal/mol)	Primer 2 ΔG (kcal/mol)
**1. Klebsiella pneumoniae KPC-1**								
AAC(6')-Ib_fwd	AAC(6')-Ib_rev	112	0	0	57.9	57.0	-13.1	-12.8
KPC-2_rev	KPC-2_fwd	101	0	0	50.3	54.0	-9.9	-11.4
SHV_rev	SHV_fwd	127	0	0	57.0	55.8	-12.7	-12.2
**2. Acinetobacter baumannii NCTC 13305**								
OXA-58_fwd	OXA-58_rev	142	0	0	51.7	56.8	-10.4	-12.8
**3. Enterococcus faecalis NCTC 13379**								
ermb_rev	ermB_fwd	114	0	0	51.6	51.7	-10.3	-10.2
vanB_fwd	vanB_rev	122	0	0	51.8	49.8	-10.3	-9.8
**4. Pseudomonas aeruginosa NCTC 13437**								
AAD_rev	AAD_fwd	84	0	0	57.7	50.2	-13.0	-9.8
AAD_rev	OXA-10_fwd	823	0	0	57.7	57.6	-13.0	-13.1
OXA-10_rev	OXA-10_fwd	150	0	0	56.1	57.6	-12.5	-13.1
VEB_fwd	VEB_rev	168	0	0	51.4	52.6	-10.3	-10.6
VIM_rev	VIM_fwd	86	0	1	56.0	54.7	-12.3	-11.7
**5. Klebsiella pneumoniae NCTC 13439**								
AAC(6')-Ib_rev	AAC(6')-Ib_fwd	112	0	0	57.0	57.9	-12.8	-13.1
AAC(6')-Ib_rev	VIM_fwd	1048	0	0	57.0	56.2	-12.8	-12.4
qnrS_rev	qnrS_fwd	121	0/1	0/1	49.6/41.9	51.9/43.0	-9.7/-7.0	-10.5/-7.1
SHV_fwd	SHV_rev	127	0	0	55.8	57.0	-12.2	-12.7
VIM_rev	VIM_fwd	86	0	0	56.0	56.2	-12.3	-12.4
**6. Pseudomonas aeruginosa M/7298**								
AAC(6')-Ib_rev	AAC(6')-Ib_fwd	112	0	0	57.0	57.9	-12.8	-13.1
OXA-10_rev	AAC(6')-Ib_fwd	1184	0	0	56.1	57.9	-12.5	-13.1
OXA-10_rev	OXA-10_fwd	150	0	0	56.1	57.6	-12.5	-13.1
SPM-1_fwd	SPM-1_rev	145	0	0	50.6	57.3	-10.1	-13.0
**7. Klebsiella pneumoniae DSM 30104**								
SHV_rev	SHV_fwd	127	0	0	57.0	55.8	-12.7	-12.2
**8. Kluyvera ascorbata DSM 4611**								
CTX-M-2_fwd	CTX-M-2_rev	124	0	0	53.8	54.9	-11.4	-11.8
**9. Enterococcus faecalis NCTC 12201**								
ermB_fwd	ermb_rev	114	0	0	51.7	51.6	-10.2	-10.3
vanA_fwd	vanA_rev	150	0/1	0	51.2/45.7	48.8	-10.3/-8.2	-8.6
**10. Acinetobacter baumannii M/10639**								
OXA-23_fwd	OXA-23_rev	102	0	0	48.5	56.0	-8.2	-12.3
PER_fwd	PER_rev	140	0	0	53.2	51.8	-11.0	-10.3

Supplementary Table S8: Predicted products and thermodynamic data at 3 mM $MgCl_2$. Original data of the PRIMEval run at max. 1 mismatch can be found under https://primeval.ait.ac.at/run/1149227354.

Primer 1	Primer 2	Size	MM 1	MM 2	Primer 1 Tm (°C)	Primer 2 Tm (°C)	Primer 1 ΔG (kcal/mol)	Primer 2 ΔG (kcal/mol)
**1. Klebsiella pneumoniae KPC-1**								
AAC(6')-Ib_fwd	AAC(6')-Ib_rev	112	0	0	59.9	59.0	-13.9	-13.7
KPC-2_rev	KPC-2_fwd	101	0	0	52.3	56.0	-10.7	-12.3
SHV_rev	SHV_fwd	127	0	0	59.0	57.8	-13.5	-13.0
**2. Acinetobacter baumannii NCTC 13305**								
OXA-58_fwd	OXA-58_rev	142	0	0	53.7	59.0	-11.3	-13.9
**3. Enterococcus faecalis NCTC 13379**								
ermb_rev	ermB_fwd	114	0	0	53.7	53.8	-11.3	-11.3
vanB_fwd	vanB_rev	122	0	0	53.8	51.8	-11.3	-10.6
**4. Pseudomonas aeruginosa NCTC 13437**								
AAD_rev	AAD_fwd	84	0	0	59.6	52.1	-13.8	-10.6
AAD_rev	OXA-10_fwd	823	0	0	59.6	59.7	-13.8	-14.1
OXA-10_rev	OXA-10_fwd	150	0	0	58.2	59.7	-13.6	-14.1
VEB_fwd	VEB_rev	168	0	0	53.4	54.7	-11.2	-11.7
VIM_rev	VIM_fwd	86	0	1	57.9	56.6	-13.1	-12.5
**5. Klebsiella pneumoniae NCTC 13439**								
AAC(6')-Ib_rev	AAC(6')-Ib_fwd	112	0	0	59.0	59.9	-13.7	-13.9
AAC(6')-Ib_rev	VIM_fwd	1048	0	0	59.0	58.3	-13.7	-13.2
qnrS_rev	qnrS_fwd	121	0/1	0/1	51.7/44.0	53.9/45.1	-10.5/-7.8	-11.3/-8.0
SHV_fwd	SHV_rev	127	0	0	57.8	59.0	-13.0	-13.5
VIM_rev	VIM_fwd	86	0	0	57.9	58.3	-13.1	-13.2
**6. Pseudomonas aeruginosa M/7298**								
AAC(6')-Ib_rev	AAC(6')-Ib_fwd	112	0	0	59.0	59.9	-13.7	-13.9
OXA-10_rev	AAC(6')-Ib_fwd	1184	0	0	58.2	59.9	-13.6	-13.9
OXA-10_rev	OXA-10_fwd	150	0	0	58.2	59.7	-13.6	-14.1
SPM-1_fwd	SPM-1_rev	145	0	0	52.6	59.4	-10.9	-14.1
**7. Klebsiella pneumoniae DSM 30104**								
SHV_rev	SHV_fwd	127	0	0	59.0	57.8	-13.5	-13.0
**8. Kluyvera ascorbata DSM 4611**								
CTX-M-2_fwd	CTX-M-2_rev	124	0	0	55.9	56.9	-12.2	-12.8
**9. Enterococcus faecalis NCTC 12201**								
ermB_fwd	ermb_rev	114	0	0	53.8	53.7	-11.3	-11.3
vanA_fwd	vanA_rev	150	0/1	0	53.2/47.6	50.8	-11.1/-9.0	-9.7
**10. Acinetobacter baumannii M/10639**								
OXA-23_fwd	OXA-23_rev	102	0	0	50.5	58.0	-9.4	-13.3
PER_fwd	PER_rev	140	0	0	55.2	53.9	-11.9	-11.3

Supplementary Table S9: The 45-plex antibiotic resistance genes (abr 1) primer set was designed using oli2go.

Primer	Forward sequence (5'-3')	Reverse sequence (5'-3')
CRP	GTACTACATCGTTAAAGGCTCTG	TTTCGCACGTACCCATG
acrE	GCAGCTCGATCCTATCTA	TCCATGACCAACTCTACGT
acrB	CCGGTCTGGGGATCAAG	TAGAGATTTTCACGAACG
MECA	GAAAAAAGATGGCAAAGATA	ATGAAGGTGTGCTTACAAGT
mdtN	TGGAAAGTACGCCGAAA	TTAGTTGATGGCGCACTGT
msbA	ATGAGTTGCAGAAAAACCG	TCCACAATGACACCATC
Penicillin_Binding_Protein_Ecoli	GAAAACTAACCTGACCGAAGTACA	TTGATTTTCGACACATAGCC
RlmA(II)	AAGGAAAATTTTCAAAACCGT	CTTCACCACAACCGATATCC
evgA	AATAATTATTGATGACCATCC	GTTTCCACCCGCTGAAC
mtrA	GTAGAAATCGACGTACCG	CACATCACGAGTAAACACC
tetM	GTACAAGCACAAACTCG	TGATAAACCGTTGATAAATCA
emrB	GTATCAGCCTCGCGTAT	GATAATCGGCGACAGGAT
mepR	ATACGTTAGGTTATCTTTATGCAC	GCGATAGATCAGCTTTTTACGTTC
bacA	CTGATAGCGGCAATATTGG	CCGACAATAATCATATGGCC
mdtD	TGACAGAWCTTCCCGAC	GCGGTGTTDACGATGGT
sav1866	GGGTTAATGAATATTTGGTTAG	GTACACCGTTAAAATGTAAAA
baeS	TTTGAACGTTTTTATCGCA	CCAGCGGTAACTCTACT
H-NS	GACGAAAACGGCGAAACTAAAA	TTATTGCTTGATCAGGAAATC
tolC	TTCTTATCGGCCTGAGC	TTCATTAATTTTTTCAAAGGCA
Tet-38	TTACGTTGCCATTCCTATTTA	ACCAATCATATATAACCAATTAAAG
PmrF	AATATATCGGCAGGATCTACA	TTATTCATTTTCCTTGCTGG
mdtL	GAATACGCCACCATTATG	CGCATGGGAAGGTGAAAC
FosB	TAGATGACAGCGAATTTAA	CTCAAGTGTGCCAGTATGTAA
acrD	CTTCCGTCGATAAGCAG	TGATATCGCGGAACTGT
TEM-116	CCGCATACACTATTCTCAG	TCACTCATGGTTATGGCAG
Aac3-Ik	GAGTATTGCTCTTAGGTACGA	TAACCATACTTTTTTCATTACT
ANT(4')-Ib	GCGTAATATTCGTGTGCAA	TCAGTTAAGACCGAAGC
cpxA	GTTACCCAAGCTCGATT	ATGCTGCTCAATCATCAG
patB	GGAACTCAAGCAAGTCTTTGAA	CTCTTGCAATGTTTCATCAGTC
arlS	AGAAGAATCGTTAAATATTTCT	TGTTTTAATGAGTGTATTCG
arlR	ATCATTATAATTACAGCGAAAAGTG	ATTCTTGCTAAAAGTTCTTCAA
emrR	GAAAACCACAGTATTCAGCCT	CTCGTGACCTTTTTCCGTTAAT
pmrA	AGCAAAATGACTCCCAAA	TAGCCAAATTGACCTGCTAC
ErmA	CAAGACAACGTAATAGAAATC	TTACCGCTTCTTTAGTCA
efpA	TACGGCTCATTTTTCATG	GATCGCCGACAGAGTCAG
mepA	TTAATGAGTATCTTAATGGGATT	ATAAGTTCTGGAAATTGCACAAG

norA	GTATGAGTGCTGGTATGGT	AAACTTCTGCCATAAATCCAC
sdiA	AATGATTTCTTCACCTGG	CTGTGTCTGATATTGCAATT
mgrA	CGATCAACGTGAAGTATTTATT	TGACTTTACCTAATAAGCGATTAAG
Dha1	ACGCTGGTATTGGTGTT	AAAACAACGATTAATCCTAAT
baeR	AGTTACCAATCGACGAAAACAC	CACGCAGATAATCAATGAG
mdtF	CTGCAAACGCCGGAAGA	GTGGAATAATCTTCCGC
mecI	TAGAAGAAATACAAATGCAAA	TTTATTGTCTTTTTTACGATCT
Spc	TTGGTTCAGCAGTAAATGG	CCGTATTTCCAATCTTTCCTGAT
leuO	ACCTGCGGTCAGTAACG	CAAAAAGTTGAAATGCGC

Supplementary Table S10: The 45-plex antibiotic resistance genes (abr 2) primer set was designed using oli2go.

Primer	Forward sequence (5'-3')	Reverse sequence (5'-3')
mdtP	CGCAGCTTTATTACAGTAT	GACCGTGCGCCACTTTA
emrD	TCCAGCTATTGCCGATAT	GGGCCATAAAACAGCTG
mdsA	TTCCAGATCGATCCGCG	GAATGCGATCGAAATCC
Aac6-Iaa	GGCATCATTTRTTGCGAT	GAACAAAAATACCTTCAAGRAA
CatA1	CAATCCCTGGGTGAGTTTC	ATCAGCACCTTGTCGCC
MphC	ACGAATGAAAGAGCATATAATAG	CTACTCTTTCATACCTAACTC
gadW	AAAAATTTGGCAGACGATTTT	GGGGKGACTGGTTAATTAA
acrF	AGAGGGTAAAGGTGTTGT	GCMCCGTTACTGATAGC
golS	ATGAACATCGGTAAAGCAGC	ACATCAGCCTGGGTATAG
PmrE	TTCTAATCGCWCAAAATCA	AGATATCCGATCATTCAGCATAG
SulII	TATTCGCGGTTTTCCAGAC	CCCGTCTTGCACCGAATG
Aph3-III	ATCACCGGAATTGAAAAAACTGA	TTTTTAAATATAGGTTTTCATTTTC
AmpC1_Ecoli	TGGAGATGATCAAGCGCAC	AAATCGACTCTTCAACATAG
evgS	GTGACCATTGACACATG	GGAATGATATTTGGTTTTCTAA
marA	TTTAAAAAAGAAACCGGTCATTC	GGTTCGGGTCAGAGTTT
arnA	AAGTACCGTAAGCGAATCATC	CGGGCCGACGATTAAATTAG
SulI	GCCGATGAGATCAGACGTA	AATCCTTGGATATCGTTCAGGT
ErmB	GGTAACGTCTATTGAATTAGA	ACCTCTGTTTGTTAGGGAATT
Aac6-Aph2	CAAAAGAAAAAGCAATATATAATTT	GTTCCTTTAATTTCTTTATAACC
Aac6-Ib	CTAAATCGATCTCATATCGTCG	CCAATCGGCTCTCCATTCA
aadA	CGTTATCCRGCTAAGCG	TCAATGTCGATCRTGGCTG
mdtB	TKTTTATTATGCGTCCTGT	GATARTCCACTTCCGGCAG
StrB	CGACTATCTGGTATGGCGC	CGGCAACGATGTGAGAG
mdtA	ACAARGTCAGCAAACATCT	TCGCCCGCAGAAATACCT
mfd	GAGCGCGAACTGGAACG	CGATCCCGGTTTCGATAATG
mdsC	CCAGCGCGTCTCTTAGC	CGATTTCAATCTGTTTTTGGACAT
acrS	CCAAAATTCCCCGCCAG	CCCATCTTTTCGCGTATCAC
mdtE	CGCACTCGTTACCGCTAAT	TGGCGACCTCTTCTTTCAT
emrA	GAGACTCAAACCCCGCAG	TAAATCCCTATCGCTACGG
emrY	CGGTTTGCTATTACTGGCG	TGTTAAGGGTAAAAAGAAACA
gadX	TAYGCAAGACATAAATATATTCT	YGAATTTGGCTTGCATC
mdtH	GTTGATAATGTCATTAAGCATG	GCACTTAAGGTTTCACGCG
AmpC2_Ecoli	CGATATTGTGCATCGCA	ACTCAAACAACGTTTGC
OXA-66	GGTACCCAAGTCGATAATTT	TTCCATTCTTTTCTTCTATGAAT
gadE	TATGGTAAACACTTGCCCCAT	GACGTGATATTGCTTTCATTT
acrA_Escherichia	TCAAAACTGAACCTCTGCA	TACCTTCTTTGAAATTACGCT

mdtG	TCACCCTGTGRAAATGA	CSGTAAGAAAACAGCCTAG
tet(A)	TTTGCTCCTTGGCTTGGAA	GCACTTGAAAAAGCCAGCAAT
mdfA	CAGTCGCCGATTATCAT	ATCAGCGAACGTACGGT
PmrC	ACGTCGACTATATTGTTGAT	GCAGATAGATGCCATTTTC
mdsB	CGCCTATGTCATTACTCT	CTCGACAATCAAAATGGC
SHV-12	RTCTGAGCGCCCGTTC	GGTCTTATCGGCGATAAAC
mdtO	CAACTGGACAGCGAAGA	CGTTATCCAGCGCCTGC
MsrA	GTAAAGCTAAACGAAATCAAGCG	TTTTCAATATGCTTAGCTTGTT
mdtM	GGTTATGTCACGGTGCA	CAAAAAGGACTTTCCAGTG

Supplementary Table S11: The 45-plex antibiotic resistance genes (abr 3) primer set was designed using oli2go.

Primer	Forward sequence (5'-3')	Reverse sequence (5'-3')
mel	GCCATGACCGCTATTTTCTTG	TCTTACGTTCTTCCTCTTT
adeI	AAGATGGTTCTACCTATCC	ATTCGGGTTAGAGAATACG
tet(D)	TAACATCCAATTAAGATATGATG	ACTGATCTAAATTATGTTCAAT
adeJ	TGTACTYGGTGGCGTAC	GATTTATGCTCCTGAGTGTTTA
oqxA	CCGCTAAGGTGCTGGTG	ATTCACTTTATCAATGTATCCC
farB	GTCGTATTTTATTGTTTGGGAAT	AGCGTCCCCATATACACCA
ADC-2	TCTATGAATAAACCTTTCGA	CGGCTGATTTTCTTGGTTATA
adeS	CGATTTATTTAAGCCTTTCT	ACTTTTCGAGCCTTGATT
MphD	TCAGATGAATATGGCTTATGT	CAAGATGCCGTTTTAAAG
LsaA	AAAAATTAAGAGATGCTTTTGAA	CGACCATTCCGCTTTTTTA
macB	GATCCGTTGGGTAAAACC	ATAGGGCGACCAAAGCA
vgaC	GGAGATCGTCCGGGAAGG	TCCGCCATAAAGTCCGG
mtrE	AGCTGATCGACATCGCAC	GTGGGCAGGAGGTTGTTG
adeG	GTCCAATGTTCTCAAGTTAT	CGTACTGAGAACCTAAATAAATCT
mtrC	GGAATCGCTGCGTACCG	TGCTTCATAAGTGGAACTGT
dfrC	TCACTAACCAAGCTTCATT	ATCTACCTGGTCAATCAT
adeB	GGATTAACTCTCCAAATAATC	CCAAATACGCATAGCTTT
MsrE	ACTGGGCGGAATCGAAATA	TTTTTGCACGAGTTTCCT
CTX-M-15	CTGTTGTTAGGAAGTGTGCC	GCACGATAAAGTATTTGCGAAT
floR	TCGGATTCAGCTTTGCCT	ATCCTGCGATGCCCCAT
adeC	TTGGGGAACAAGAAAAAC	TGYGCATGTGTAGCAAG
KPC-3	GCTGTCTTGTCTCTCAT	CTTACAGTTGCGCCTGA
macA	TATGCCCGTTCGTTTGTG	GCGATTTTTCACGGTCAG
adeF	GTATTCAACGTCTCATTCA	ATTGGACAGCAGCTCTAG
mphD	AAATCCATAGTATTCCTGAAAAA	TTCAGATTTTACTAACTGCAAAT
mtrD	CGTCCGATTATCATGACCTC	CATCCCCCAGAATACGGT
BlaA1	CTTCATGCTTGGGACGA	GAACGCCACCAATAATTTTCA
mtrR	CTGGCGCGAGAAAATTAC	CCAAATCAGCCCGTCCAA
Sat-2A	ATGAAGATTTCGGTGATCCCTG	ACTTCTGGTAGATAGTTCAA
CMY-2	CGTTGAGGTAAACCCGCC	CCAAGGTTTTTTTCTGGAACGAA
OXA-23	AGAATATGTGCCAGCCTCTA	CCCTTCCATTTAAATATTTCAT
Mbl	CACGTYTAGGTCAGAATCG	GTGAARTTGAGCCGCAA
adeK	CAAAAGCACGTTTATTCCC	CCCCAGTCAAAGATTGG
oqxB	CCCTACCCGGCTGATCG	CCCTGATAGCCGTTCGA
Mrx	CGACCTTTCTCTTCCGGAT	GATGATGGTCGCGATGA
ErmC	TTTTAATGGCAGAAGTTGA	GAGCTATTCACTTTAGGTTT

APH(3′)-Ia	AACGGTTTGGTTGATGC	CTGAATCCGGTGAGAATGGC
abeS	ATCGGGATTGCCTATGCC	AGGCAGCYAAGTCTAAAT
MefA	GGTCTTGTCTATGGCTTCAYTA	GCTGCTGCGATAATTAAATC
FosA5	ACTGAATCACCTGACCC	ATCGCCGCAGGAGAGATA
adeA	CACATTCCGTATTGAAGTTAATAAC	CTCGCCACTGATATTACGTTG
acrA_Klebsiella	GGCCTGCAAAAAATCAAAC	TTGTTCTGATGGCGCGT
adeR	CGGCGCTAGATCAAGATA	CTGCCTGAACTCTAGCGA
PDC-1	GGTGATGAAGGCCAATGAC	TCTTTCGAGGCCAGCCC
mphA	CCAACTGTACGCACTTGCA	ACCCACCGACGTCCATC

Supplementary Table S12: The 45-plex antibiotic resistance genes (abr 4) primer set was designed using oli2go.

Primer	Forward sequence (5'-3')	Reverse sequence (5'-3')
MexA	AACAACGAGCTGCTGCC	GTAGCCTGGCCCTTGAG
Zn-dependent_hydrolase	CACATTCTTTACAGTTTGAT	CTACCCATTGTTGCTTATTT
aad(6)	TATTGAAAGGTATATATCCGAGGA	CTCCGGATAGGCATAATGAA
mexK	CATGATCATGCGCAACTC	CCTCGATGATGGCGTGC
CpxR	CAGATGCAACTGGGCGAC	AGGAGCGCTTCGAGGAT
MexE	GGAAACCACCCAAGGCAT	TCGATGTAGCCCGAYAC
AAC(6')-Ii	TCAATTACTTAGAAAAAGAAGT	GATGTTCACGAAGGTTCT
MexF	GGCATCACCGTCGACAAG	GGCCAGTTCGTCCTTCAC
NDM-1	TGACAATATCACCGTTGG	GTAGTGCTCAGTGTCGGC
AadA5	GTCGGATTGGAAGGGTG	CAAACGCTCCGATACCCA
mexW	TGATCATGGTCACCGTG	ACTCGACGATCAGGATGC
mexP	TGTCGGGCATATCCAGG	GTGCGCTCGTCGACATC
OXA-9	TTCCGCCACTCTCCCAAT	GTATTTCCGGTAATGGCG
TriC	ATGAACAGCGTGGTGACC	GACGATCTGCAGGCTCT
mexG	GGACCGCAAGCTATGGC	CAGCTTGGCTTCGACGC
mexQ	ATCGTTCTTTCGGTGCTG	GATCACGTCGGGGTTGG
OprJ	CAGCAGCTACCAGGTCG	GTCGGTCAGGCTCTTGAC
MexB	CGTGGTCCAGGTGATCG	TGCCGTCGGAGTTACTCT
mexV	GTTGATCATGCTCGCCG	CTGCCAGGGACGCTTTTC
MuxB	CTGTTCATGGGCGACGT	CTGCTGGTCCTCGTCGA
TriB	GACGGCAAGACCCAGGT	AGCACCTGTACCTCGCG
CatB7	AGCTGCTCTCGGAACAG	TGACCAGCTTGTCCACGT
CARB-8	TACTGATTTTTTAAGACAAATTGGG	TTGCCTTAGGAGTTGTCGTAT
adeN	GATGTCCAAAATACAATTGC	ATAATCATTCGCCATTCAATATC
msrC	GAGGGGAAGAACGAAAG	CTTTTTCTATCTAGATGGGT
amrA	CTGGTCGGCGAGGACTC	CCCTTCACCTGGCCTTC
OpmH	CCGTGCGCGACTACAAC	TCAGGTAGGCGCTGAGC
OXA-50	GTCTCGCGCCTGGGTTA	GCGGGGAATGGCAATTCT
OpmB	CTGCTCAACGACACGGT	GGGCCTGGGTGCTTTTC
mexJ	AGACGGTCTTCAGCCTG	GCGTTGCGACCAGAGTTC
OprM	TCGATCAACCTGCCGATCT	ATCGCCTTCTCGTACTG
opmD	CTGTTCGACGAYCGCTG	AGGTCGATTTCCCACTGC
mexI	GACCATCCGCATGCTGC	GTGGTCTCGCCGGAAGA
arnA	TCGAGTACACGCGCAAC	CCTCGGAGGTGGAAGGG
amrB	TCGATCAGGAAGGTGGTC	CGAGCATCACCGTGAAGG
DfrA12	TAGTTGTTTCAACGCTGTCG	GTCACCCTCGAAGGTTTGAT

farA	GGCATATGAAAATCGGAC	GCCGGAATCAGCGAAAA
Aph3-IIb	CTGCCCGCCGAAATCG	CTCATCAGCAGCCATTG
VIM-2	ATTGTCCGTGATGGTGA	GAGACTGCACGCGTTAC
MuxC	CGACCATCTTCAACCCGC	CGATCACCTGGACCTGG
opmE	GAGGTACGCGGCTCGAT	CAGCGAATCACCGGGTT
basS	AGATCTGGATCAGCGAAAA	CAACGCCGAACCAGACC
MexD	CTGGTGGACTCCGTCAC	GAAGGTGACGACGATCTC
AphA6	CGAAAATGTTGAGTTGGCTC	TTTTGCATTGATCGCTTT
mexL	AAGCCATCGCCTCGGAA	TGGAAGTACAGGGCGGG

Supplementary Table S13: Bacterial genomes used for the in silico evaluation of PRIMEval. Strains marked with (*) were sequenced in-house on an Ion Torrent Personal Genome Machine (PGM).

Species	Strain designation	ENA accession
Abiotrophia defectiva	DSM 9849	ACIN03000001
Acinetobacter baumannii	DSM 30007	JMRY01000001
Acinetobacter baumannii	NCTC 13305	GCA_900444775
Acinetobacter baumannii (*)	41_ACB	ERS3407745
Acinetobacter baumannii (*)	42_ACB	ERS3407746
Acinetobacter baumannii (*)	43_ACB	ERS3407747
Acinetobacter baumannii (*)	44_ACB	ERS3407748
Acinetobacter baumannii (*)	5M	ERS3407749
Acinetobacter baumannii (*)	IMP	ERS3407750
Acinetobacter baumannii (*)	M/10639	ERS3407751
Acinetobacter calcoaceticus	DSM 30006	APQI01000000
Acinetobacter lwoffii	DSM 2403	CP000139
Acinetobacter nosocomialis	DSM 102856	APOP01000001
Bacteroides fragilis (*)	M/7021	ERS3407752
Bacteroides vulgatus	DSM 1447	CP000139
Campylobacter coli	NCTC 11366	GCA_900446355
Campylobacter lari	NCTC 11352	GCA_900446615
Chromobacterium violaceum	DSM 30191	AE016825
Citrobacter amalonaticus	DSM 4593	GCA_900460855
Citrobacter koseri	DSM 4595	GCA_900446925
Clostridium perfringens	DSM 756	CP000246
Cronobacter sakazakii	DSM 4485	GCA_900447395
Enterobacter aerogenes	DSM 30053	CP002824
Enterobacter cloacae	DSM 30054	GCA_900447605
Enterobacter cloacae (*)	8EC	ERS3407754
Enterobacter cloacae (*)	17604	GCA_900465085
Enterobacter cloacae (*)	30676	GCA_900465105
Enterobacter cloacae complex (*)	NCTC 13925	ERS3407755
Enterobacter hormaechei ssp. oharae	DSM 16687	CP017180
Enterococcus faecalis	ATCC 29212	GCA_900447845
Enterococcus faecalis	NCTC 12201	GCA_900447835
Enterococcus faecalis	NCTC 13379	GCA_900448045
Enterococcus faecium	ATCC 700221	CP014449
Enterococcus faecium	DSM 20477	GCA_900447735
Enterococcus hirae	DSM 20160	JXKR01000001
Escherichia coli	DSM 30083	KK583188
Escherichia coli	NCTC 13462	GCA_900448525
Escherichia coli	DSM 105182	GCA_900448335
Escherichia coli (*)	15E	ERS3407756
Escherichia coli (*)	23E	ERS3407757
Escherichia coli (*)	24E	ERS3407758
Escherichia coli (*)	31E	ERS3407759
Escherichia coli (*)	7E	ERS3407760
Escherichia coli (*)	8E	ERS3407761
Fusobacterium nucleatum	DSM 15643	AE009951
Fusobacterium ulcerans	DSM 19847	ACDH02000001
Granulicatella adiacens	DSM 9848	ACKZ01000001

Haemophilus aegyptius	DSM 21187	AFBC01000001
Haemophilus influenzae	DSM 4690	GCA_001457655
Klebsiella aerogenes (*)	122664	ERS3407753
Klebsiella pneumoniae	DSM 30104	AJJI01000001
Klebsiella pneumoniae	NCTC 13439	GCA_900451545
Klebsiella pneumoniae	NCTC 13465	GCA_900461465
Klebsiella pneumoniae (*)	6-M	ERS3407762
Klebsiella pneumoniae (*)	KPC-1	ERS3407763
Klebsiella pneumoniae (*)	OS_2	GCA_900465095
Klebsiella pneumoniae (*)	OS_8	GCA_900465155
Moraxella catarrhalis	DSM 9143	LWAH01000000
Moraxella osloensis	DSM 6998	CP014234
Mycobacterium abscessus	DSM 44196	CU458896
Mycobacterium intracellulare	DSM 43223	CP003322
Pantoea agglomerans	DSM 3493	GCA_900454405
Parabacteroides distasonis	DSM 20701	CP000140
Prevotella bivia	DSM 20514	AJVZ01000001
Prevotella intermedia	DSM 20706	AUTZ01000001
Proteus mirabilis	ATCC 14153	GCA_900455195
Proteus mirabilis (*)	P 1	ERS3407764
Proteus penneri	DSM 4544	GCA_900455015
Providencia rettgeri	DSM 4542	GCA_900455085
Pseudomonas aeruginosa	NCTC 13437	GCA_900706955
Pseudomonas aeruginosa	DSM 50071	LN831024
Pseudomonas fluorescens	SBW25	AM181176
Pseudomonas otitidis	DSM 17224	FOJP01000001
Pseudomonas putida	BIRD-1	CP002290
Pseudomonas stutzeri	DSM 5190	CP002881
Pseudomonas stutzeri	DSM 4166	CP002622
Salmonella bongori	NCTC 12419	GCA_900635485
Salmonella enterica sv. typhi	ATCC 19430	AE014613
Salmonella enterica sv. typhimurium	DSM 554	AE006468
Serratia marcescens	DSM 30121	GCA_900457055
Sphingomonas paucimobilis	DSM 1098	GCA_900457515
Staphylococcus aureus	USA300-JE2	CP000255
Staphylococcus aureus (*)	M/8912	ERS3407765
Staphylococcus epidermidis	ATCC 14990	CM007846
Staphylococcus haemolyticus	DSM 20263	GCA_900458595
Streptococcus dysgalactiae subsp. dysgalactiae	DSM 20662	GCA_900459225
Streptococcus mitis	DSM 12643	GCA_000148585
Streptococcus pneumoniae	ATCC 49619	AP018938
Streptococcus pyogenes	DSM 20565	ATXR01000001
Streptococcus sanguinis	DSM 20567	GCA_900475505
Yersinia enterocolitica	DSM 4780	ERR731666

Supplementary Table S14: The 45-plex primer set targeting the most common antibiotic resistance genes used for experimental evaluation was designed using oli2go.

Primer	Forward sequence (5'-3')	Reverse sequence (5'-3')
AAC(6')-Ib	GCCCAGTCGTACGTTGC	CTTTGCCCAGTTGTGATGC
AAD	GATGAGCGAAATGTAGTG	GGCAGCGACATCCTTCG
ACC	CCGATTGTTCCCCCGTTA	GATTGAGTAGTTTTTGTTAGCCA
ACT	GGATGAGGTCACGGATA	CCAAAAAGACCGATGCT
aph2_consensus	TTTRTGCTTGCTGGATTGTA	TGCTTTTCTTTCTGCTACTTC
CcrA_consensus	CACAGAAAAGCGTAAAAATA	GCGAGGGATACATAAGT
cepA	AGACTTATACATTTATCCATTATC	TATTTCGGCTTTTTTCCC
CMY	TATGTACCAGGGATTAGGCTG	CTCAACGGCGGGAAGC
CTX-M-1	AGGAAGTGTGCCGCTGT	AATTATCTGCTGTGTTAATCA
CTX-M-2	TGCCGAAATCATGGGTAG	GGTTGGGTAAAGTAGGTCAC
CTX-M-25	CAGTAAAGTGATGGCGGTA	AATGGGGTTGTAGTTAATC
CTX-M-9	ACTACGGCACCACCAATG	TGCGGCTGGGTAAAATAG
DHA	TAAGCCCTATTATTTCAATTATG	CTCTTTTTTCGCCACAG
DIM	GTACATTGGATTCGTAAAAAT	CGTAGAAATTGATTGGTCA
ermB	ATCTATTCAACTTATCGTCAGA	TCCCAACAATTTTATACCTCT
ermF	AATTAGAACCTACACAAAAGTTAT	ACCTACCTCATAGACAAG
FOX	SAAAGGTTCCGCCTT	GCATCTTGTCATTCGAATC
GES	CACCTCGACCCACACCA	CCAACAACCCAATCTTT
GIM	AAGTTATAAAAATTGAAGATGGAGT	AAGGTGTGTCGATAATATAGGCT
IMI_consensus	GYAAATCATTTTCRTACARAGCA	GTGATGGGTGAATRGAAC
IMP	GGAATAGAGTGGCTTAATT	AACTTCAATTTTATTTTTAACTAG
KPC-2	TCTAGTTCTGCTGTCTTGTC	AGTCCTGTTCGAGTTTAG
MCR-1	CTCCAAAATGCCCTACAGA	CTTAACAAAAGCCACAAG
mecA	AACAAGTTCCAGATTACAAC	GCCAACCTTTACCATCG
mecC	GTTCCATACCATTAGTTAATATAT	GCTTTAGAAAATAACGGAAATAT
MOX	TGCACCACACCTATGTC	GCTTGTCCTCTTTCGA
NDM	CAGCACACTTCCTATCTCG	GGTCCAGGCGGTATCGA
OXA-1	GAAAACACCATAGAGAACA	TATAATAAACCCTTCAAACCA
OXA-10_consensus	TTTTCTGGTGTGGGAACTGAG	GATTTTGGTGGGAATGGATTTTC
OXA-23	TTCAAACAGATAAAAAAATTAATC	CGATCAGGGCATTCAACATT
OXA-24	ACTCAAGAAGAAGTTAAAAAAATG	CCAGTCAACCAACCTACCT
OXA-48	GTATCACAATAAGTTACACGT	CCAGTTTTAGCCCGAATAAT
OXA-58	TGCCTTTTAAACCTGAAGT	TTTTCAACAAAACCCACATACCA
PER	GAGTTTAGTGTTCCAGTGC	GTCTCCTTTATACCCATAGAC
qnrA	TATCAGTGTGACTTCAGCC	CCTTGAAACTGGCATCGC
qnrB	CGTTCAGTGGTTCAGATCT	AATTGGTCAGATCGCAATG

qnrS	GTGTGATTTAAAAGGKGCC	CCKCTCCATATTGGCATA
SHV	TGCAGTGGATGGTGGAC	CCAAGCAGGGCGACAAT
SPM-1	TTACAAGAAGATGGGCG	AACAGGATGGGAACTCAGAATC
TEM	CGCCGCATACACTATTCTC	GTCAGAAGTAAGTTGGC
tetQ	GTAGAAGATATGGAAGATTTTC	GACGGAGGATTTGAGAG
vanA_consensus	CGGAAAAAGGCTCWGAA	CGTTATCTTGTAAAAACATATC
vanB	GGTAAAATCCGCAATAGAAAT	CTTCCCATTCCGTACAT
VEB	TCAAGACCTTTTGCCTAAA	AAGAATTTTTGAACAGAATCAGT
VIM	GGTCTACCCGTCCAATGG	CCGCTGTGTTTTTCGCA

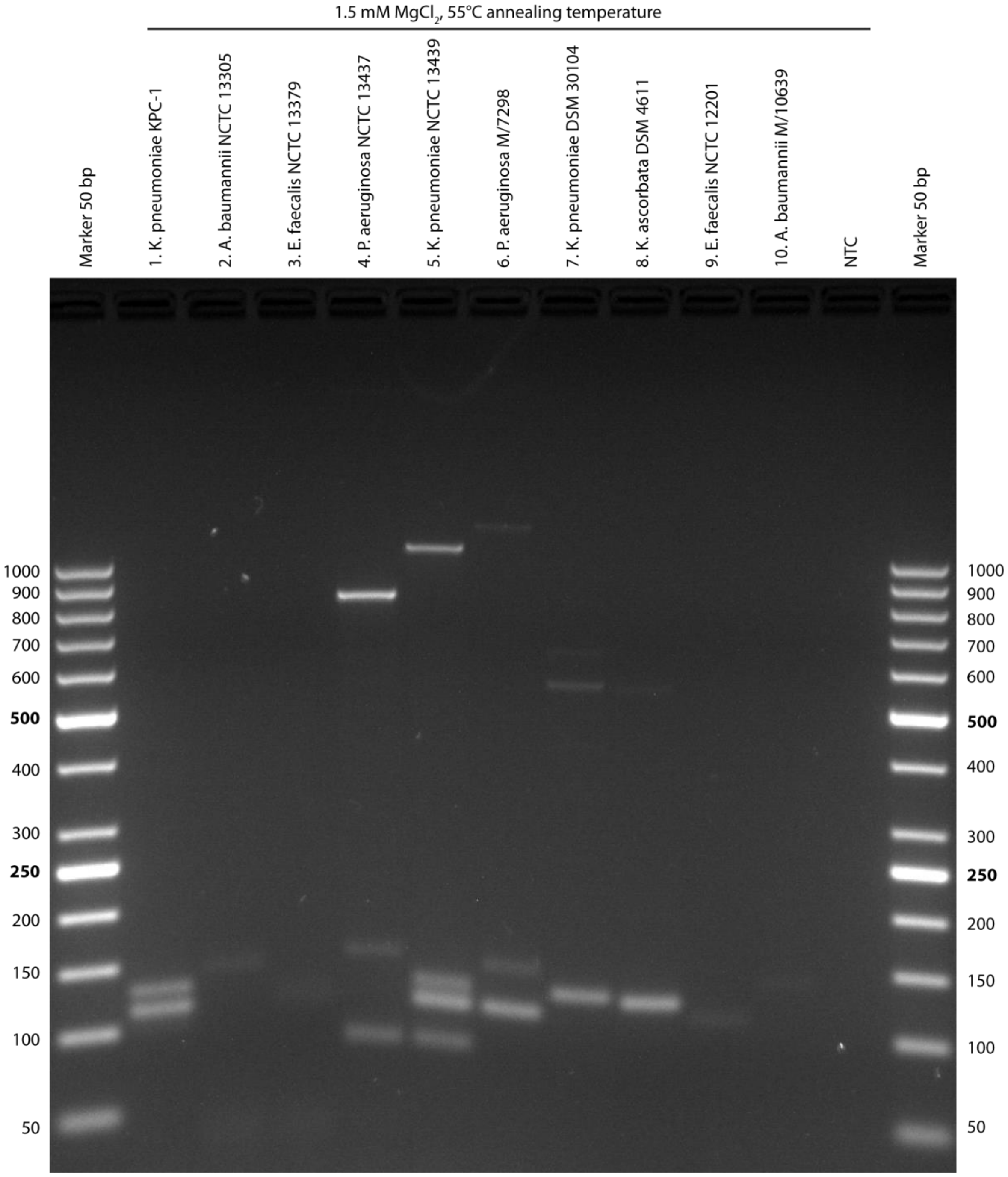

Supplementary Figure S1: The full-length agarose gel image shown in Fig. 2f (1.5 mM $MgCl_2$).

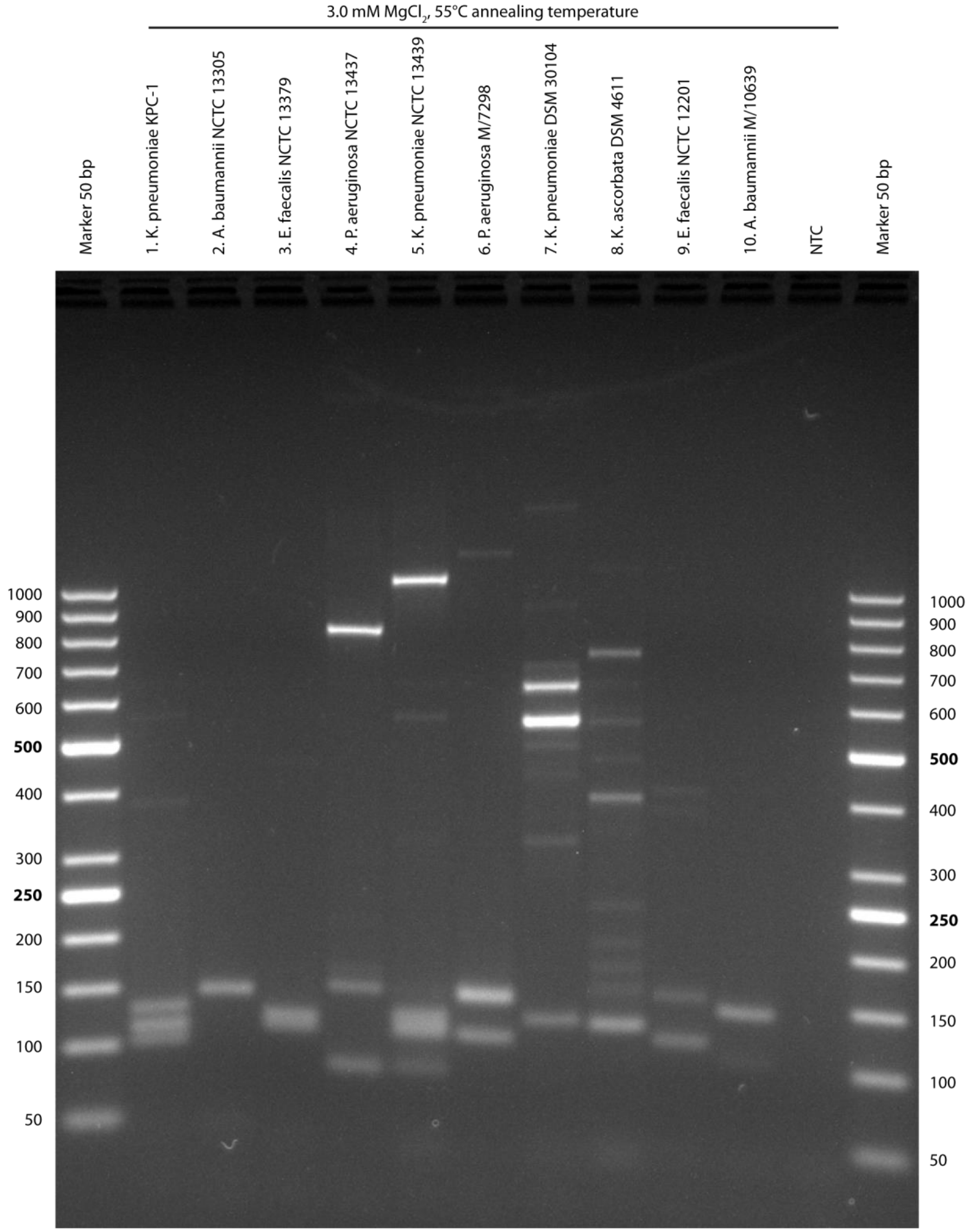

Supplementary Figure S2: The full-length agarose gel image shown in Fig. 2g (3 mM $MgCl_2$).

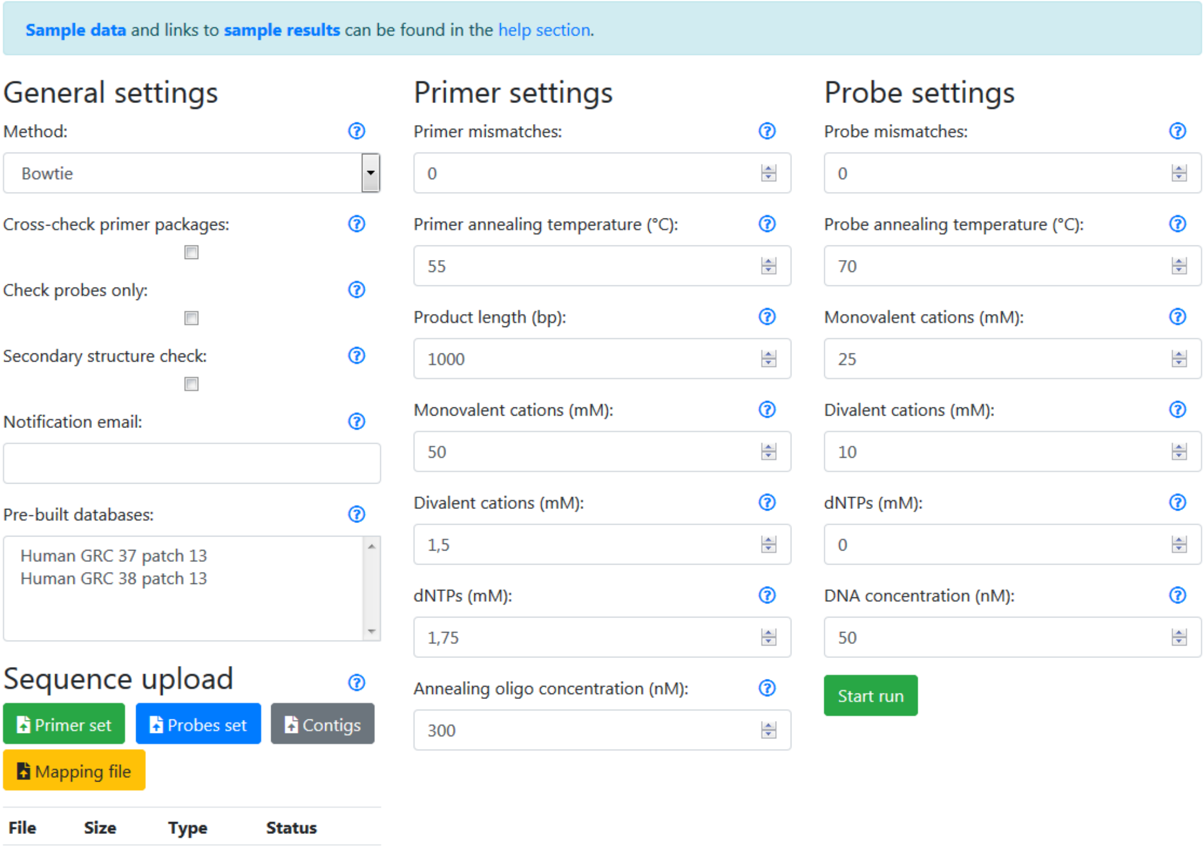

Supplementary Figure S3: The user interface of the public PRIMEval web server.

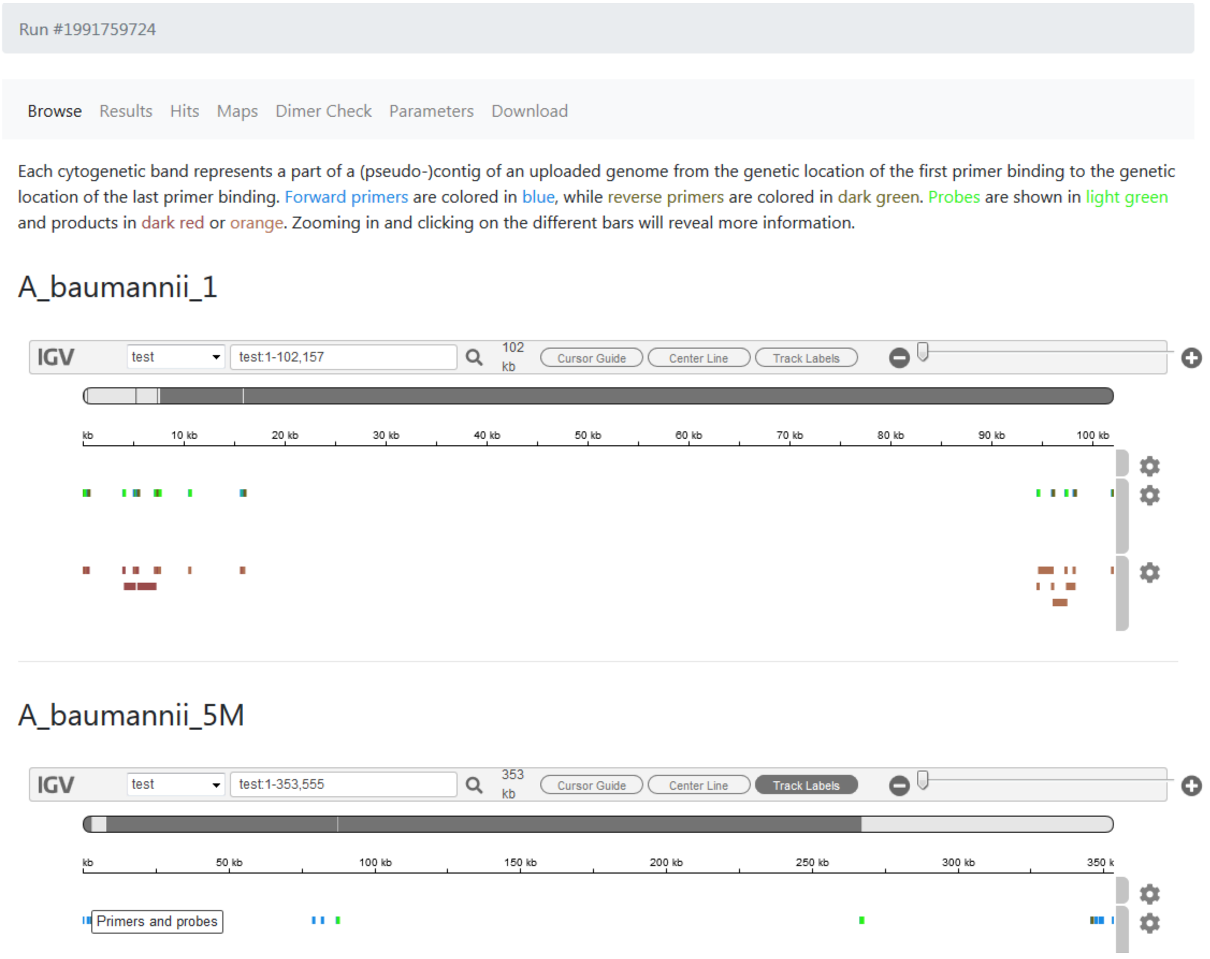

Supplementary Figure S4: The results view of the public PRIMEval web server. On the open tab (Browse) users are presented with a visual representation of their hits.
